# Phylogenetic Characterization of Fecal Microbial Communities of Dogs Fed Diets with or without Supplemental Dietary Fiber Using 454 Pyrosequencing

**DOI:** 10.1371/journal.pone.0009768

**Published:** 2010-03-22

**Authors:** Ingmar S. Middelbos, Brittany M. Vester Boler, Ani Qu, Bryan A. White, Kelly S. Swanson, George C. Fahey

**Affiliations:** 1 Department of Animal Sciences, University of Illinois, Urbana, Illinois, United States of America; 2 Division of Nutritional Sciences, University of Illinois, Urbana, Illinois, United States of America; 3 Institute for Genomic Biology, University of Illinois, Urbana, Illinois, United States of America; 4 Department of Veterinary Clinical Medicine, University of Illinois, Urbana, Illinois, United States of America; University of Wisconsin-Milwaukee, United States of America

## Abstract

**Background:**

Dogs suffer from many of the same maladies as humans that may be affected by the gut microbiome, but knowledge of the canine microbiome is incomplete. This work aimed to use 16S rDNA tag pyrosequencing to phylogenetically characterize hindgut microbiome in dogs and determine how consumption of dietary fiber affects community structure.

**Principal Findings:**

Six healthy adult dogs were used in a crossover design. A control diet without supplemental fiber and a beet pulp-supplemented (7.5%) diet were fed. Fecal DNA was extracted and the V3 hypervariable region of the microbial 16S rDNA gene amplified using primers suitable for 454-pyrosequencing. Microbial diversity was assessed on random 2000-sequence subsamples of individual and pooled DNA samples by diet. Our dataset comprised 77,771 reads with an average length of 141 nt. Individual samples contained approximately 129 OTU, with *Fusobacteria* (23 – 40% of reads), *Firmicutes* (14 – 28% of reads) and *Bacteroidetes* (31 – 34% of reads) being co-dominant phyla. Feeding dietary fiber generally decreased *Fusobacteria* and increased *Firmicutes*, but these changes were not equally apparent in all dogs. UniFrac analysis revealed that structure of the gut microbiome was affected by diet and *Firmicutes* appeared to play a strong role in by-diet clustering.

**Conclusions:**

Our data suggest three co-dominant bacterial phyla in the canine hindgut. Furthermore, a relatively small amount of dietary fiber changed the structure of the gut microbiome detectably. Our data are among the first to characterize the healthy canine gut microbiome using pyrosequencing and provide a basis for studies focused on devising dietary interventions for microbiome-associated diseases.

## Introduction

The intestinal tract of humans and animals is among the most densely populated microbial habitats on record, with estimates of densities in the colon of up to 10^12^ cells/ml [1]. Because of the large number of microbial cells in such a confined space, each with its own metabolism, the community as a whole can greatly affect the immediate environment (the host). The gut microbiome as found in animals today is likely the result of co-evolution of the host and its microbes over millions of years, and shaped by selection pressure over time [2]. These forces have led to the mutualistic host-microbe relationship, where there is benefit to both the host and the microbe to keep the environment stable.

Currently, as knowledge of the gut microbiome is being generated, it is becoming apparent that the microbiome may not always be beneficial to the host. Inflammatory bowel diseases have been associated with changes in the “healthy” gut microbiome of humans [3]–[5]. Recent observations in dogs with inflammatory bowel disease also indicate changes in the gut microbiome [6]. Furthermore, obesity may be linked to the composition of the gut microbiome [7]–[9]. Understanding of how the healthy gut microbiome is altered under disease conditions is imperative in devising potential nutritional interventions aimed at alleviating disease or disease symptoms.

Companion animals suffer from many of the same maladies as humans; however, knowledge of the gut microbiome in dogs is much less complete than that in humans. Several large clone libraries have been generated from human fecal samples and colonic biopsies, showing dominance of Firmicutes and Bacteroidetes phyla [10], [11]. Smaller clone libraries exist for the dog gut, showing co-dominance of *Firmicutes*, *Bacteroidetes*, and *Fusobacteria* [12], [13]. In addition to knowing little about the phylogenetic composition of the dog microbiome itself, even less is known about its metabolic capacity and how it is affected by external factors (e.g., diet). In mice and humans, it appears that diet can affect gut microbial composition [7], [9]. In dogs, however, a fiber-enriched diet (5% soybean hulls plus 5% beet pulp) was reported to have no effect on the microbial fingerprint as measured with denaturing gradient gel electrophoresis (DGGE) analysis, compared to a low-fiber control diet [14]. Analysis of canine microbial communities beyond DGGE profiling has been limited due to the laborious and costly methods available until recently. Next-generation sequencing techniques have decreased the cost and increased the speed of DNA sequencing, thereby allowing for deeper analysis of gut microbiome composition. The composition of microbial communities based on 16S rDNA sequence data can now be analyzed on multiple environmental samples at once utilizing 454-pyrosequencing with barcoded primers to amplify particular 16S sequences [15].

Our objectives in this experiment were to characterize the phylogeny of the canine hindgut microbiome using barcoded 454-pyrosequencing, and assess the phylogenetic changes induced by dietary fiber.

## Results

Estimated metabolizable energy intake among dogs was not different between the C and BP diets (978 and 1,024±92 kcal/d, respectively). We were able to obtain 17,828 high quality sequence reads from 5 samples of animals fed the C diet (range: 2,587 – 5,869; Table 1, Sequence Data S1), and 21,660 reads from 5 samples of animals fed the BP diet (range: 2,691 – 9,294; Table 1, Sequence Data S2). Samples from the sixth animal were not usable due to technical difficulties. From the mixed DNA samples from 6 animals on the C diet and 6 animals on the BP diet, we were able to obtain 14,022 (Sequence Data S1) and 24,261 (Sequence Data S2) sequence reads, respectively. After trimming the primer sequences, barcodes and adapter tags, the average sequence length was approximately 141 nt and the total dataset comprised approximately 10,965,700 nt.

**Table 1:** Number of sequences obtained from fecal samples from dogs fed either a low-fiber diet (C) or a fiber-supplemented diet (BP) and similarity-based species richness estimates obtained from 2000-sequence subsamples using DOTUR.

Sample	Total Sequences	Parameters calculated using 2,000-sequence subsamples		
		OTU^1^	ACE^2^ (95% CI)	Chao^3^ (95% CI)
G_C	2,707	147	249 (204 – 328)	231 (188 – 319)
G_BP	2,691	134	176 (156 – 215)	179 (153 – 238)
M_C	3,136	146	199 (175 – 242)	182 (163 – 223)
M_BP	2,900	128	163 (145 – 198)	169 (145 – 225)
O_C	3,529	134	167 (151 – 200)	160 (145 – 196)
O_BP	9,294	105	124 (113 – 148)	121 (111 – 151)
S_C	2,587	136	181 (160 – 221)	169 (151 – 211)
S_BP	3,762	112	140 (125 – 173)	156 (128 – 229)
V_C	5,869	113	135 (123 – 161)	143 (124 – 194)
V_BP	3,013	130	190 (161 – 246)	205 (163 – 301)
Mixed_C	14,022	120	157 (138 – 195)	190 (148 – 295)
Mixed_BP	24,261	136	166 (151 – 196)	179 (154 – 239)

1 Operational Taxonomical Unit at 96% similarity.

2 Abundance-based Coverage Estimator.

3 Bias-corrected Chao1 richness estimate.

**Figure 1:**
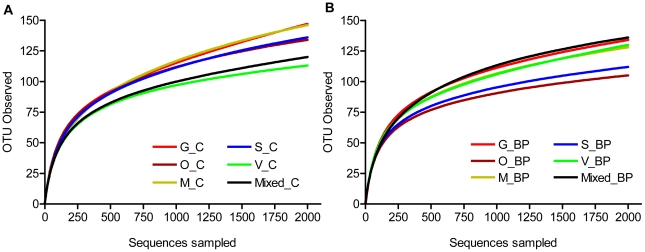
Rarefaction analysis of V3 16S data from canine fecal samples. (**A**) Dogs fed a low-fiber control diet. (**B**) Dogs fed a diet supplemented with beet pulp fiber. Each line represents a single animal or a pooled sample. Analysis was performed on a random 2,000- sequence subset from each sample. Operational Taxonomical Units (OTU) in this analysis were defined at 96% similarity.

Bacterial diversity in 2,000-sequence subsets from each sample as evaluated by rarefaction is shown in Figure 1 and Table 1. In dogs fed the C diet (Figure 1a), estimated diversity ranged from 113 – 147 OTU at 96% similarity, whereas the estimate for the pooled sample was 120 OTU. In dogs fed the BP diet (Figure 1b) the number of estimated individual OTU ranged from 105 – 134 and was 136 for the pooled sample. The ACE and Chao1 estimates of diversity (Table 1) were considerably higher (18 – 69% and 15 – 58%, respectively) than the observed number of OTU and showed non-overlapping confidence intervals among samples based on the analyses of 2,000-sequence subsets.

**Figure 2:**
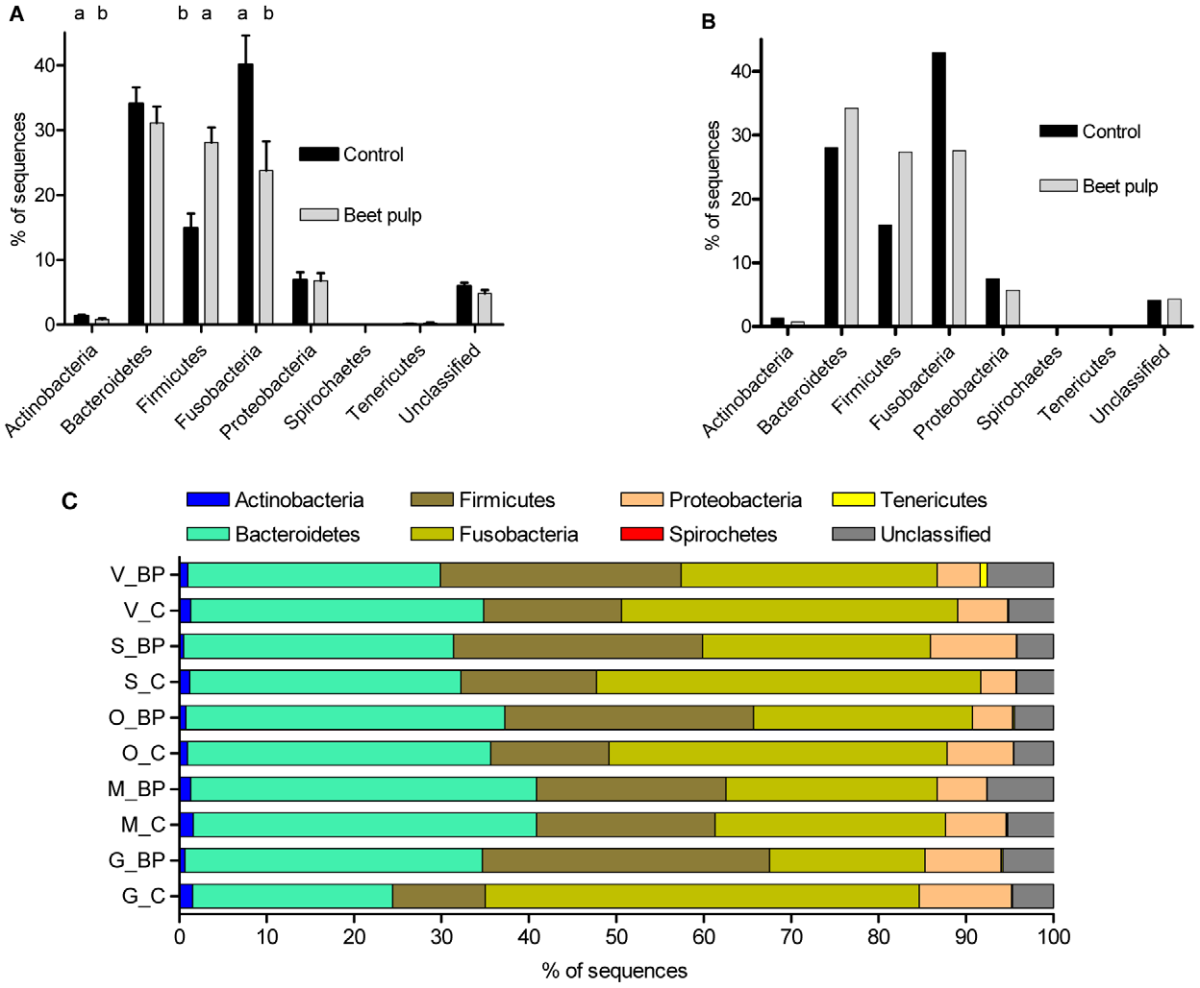
Phylum assignment of V3 16S sequences from dogs fed diets with or without supplemental fiber. Assignment according to the Ribosomal Database Project classifier (v10.2; ≥80% confidence). (**A**) Means of all individual fecal DNA samples. ^ab^Columns within phylum not sharing letters are different (P<0.05). (**B**) Observed values for single fecal DNA samples pooled by diet. (**C**) Phylum assignment of V3 16S sequences from fecal samples from individual dogs fed diets with (BP) and without (C) supplemental fiber, according to the Ribosomal Database Project classifier (v10.2; ≥80% confidence). The changes that occur in individual animals may be lost when DNA samples are pooled, or when population means are calculated.

**Figure 3:**
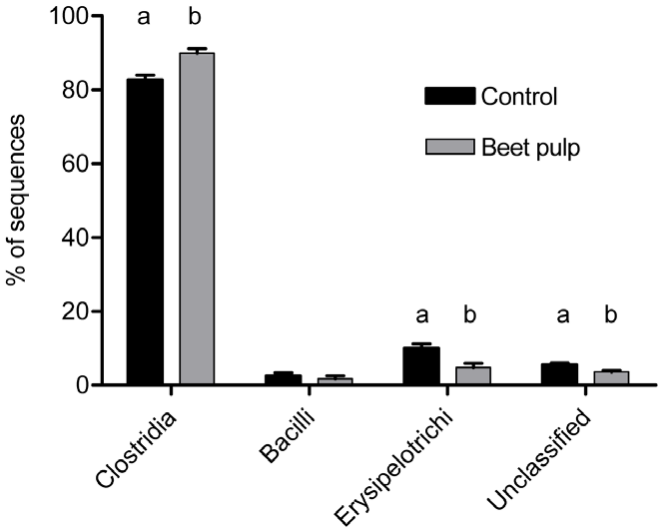
Changes within *Firmicutes* in fecal samples of dogs fed diets with and without supplemental fiber. Class assignments according to the Ribosomal Database Project classifier (v10.2; ≥80% confidence). ^ab^Columns within class not sharing letters are different (P<0.05).

Phylum sequence distribution was affected by diet. In general, a high percentage of reads across samples were assigned to Bacteria (≥96.5%) and subsequently to a phylum within Bacteria (≥89.2%). The difference between raw sequences and bacteria-assigned sequences was due mostly to short (<59 nt) reads, and a few low-confidence assignments. In the individually sequenced samples, the percentage of sequences assigned to *Actinobacteria* (1.4 to 0.8%) and *Fusobacteria* (40 to 24%) was lower (P<0.05) when dogs were fed the BP diet, whereas Firmicutes were increased (15 to 28%; P<0.05) by the BP diet (Figure 2a). With the exception of *Bacteroidetes*, the pooled DNA samples (Figure 2b) showed a similar pattern in changes of bacterial phyla as noted in the individual samples. Figure 2c illustrates that phylum distribution can be more variable in individual samples than estimates from pooled DNA samples (Figure 2b) or a statistically determined sample mean (Figure 2a) may indicate. Figure 3 demonstrates the changes within the phylum of *Firmicutes* when dogs were fed the different diets. The class of *Clostridia* is dominant (≥82% of sequences) in this phylum, regardless of diet, but still increased (83 to 90%; P<0.05) when dogs were switched to the BP diet. The increase in *Clostridia* was complemented by a decline in *Erysipelotrichi*, which were reduced by half (10 to 5%; P<0.05). *Actinobacteria* and *Fusobacteria* also changed significantly in their total representation by dietary treatment (Figure 2a); however, they were not evaluated below phylum level due to the low diversity within these phyla.

**Figure 4:**
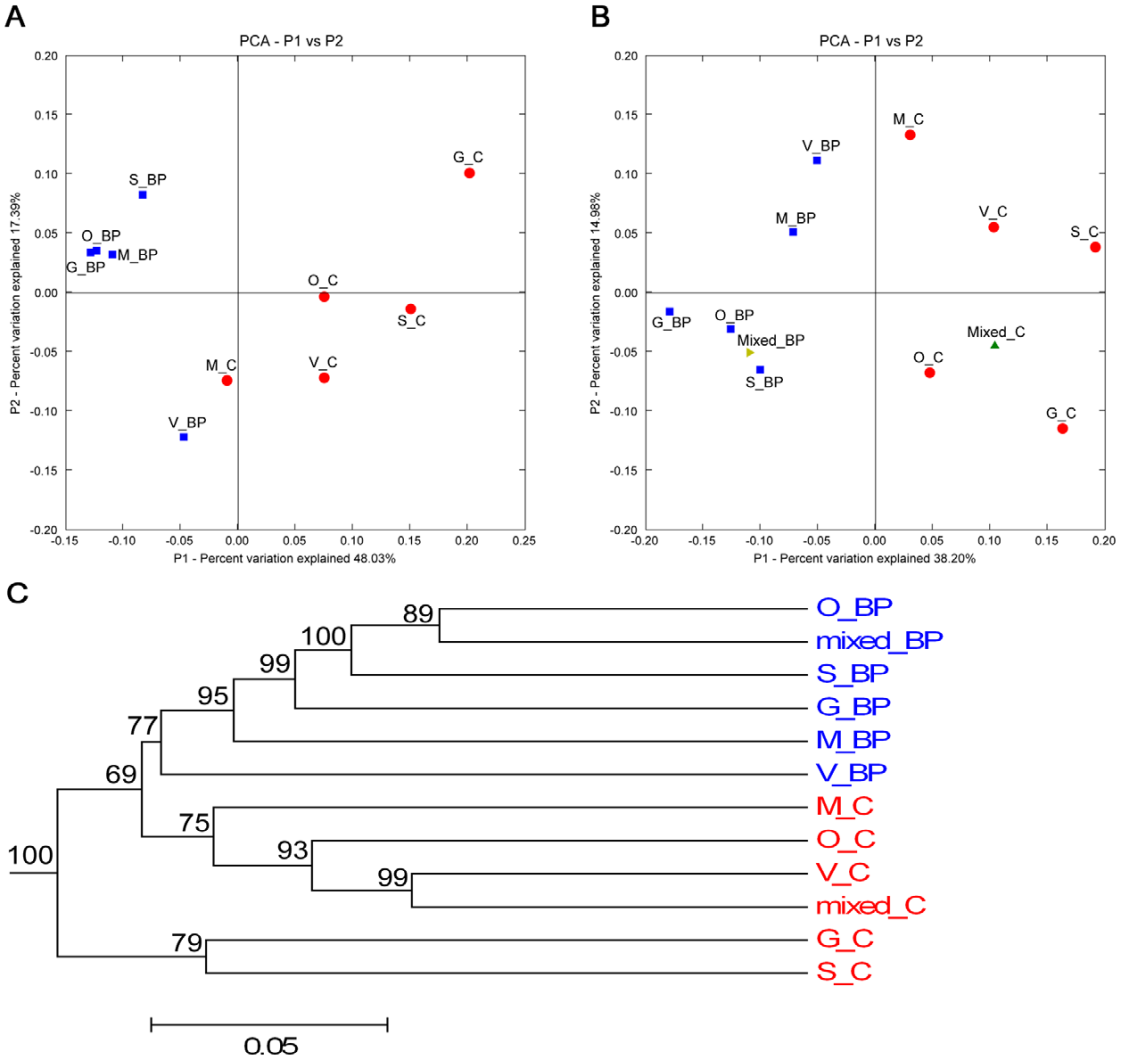
UniFrac analysis of V3 16S sequences from canine fecal samples. (**A**) Principal Component Analysis scatter plot of individual samples by dietary treatment (control  =  red circles; beet pulp supplemented  =  blue squares). (**B**) Principal Component Analysis scatter plot of individual samples combined with pooled DNA samples (pooled control  =  green triangle; pooled beet pulp  =  gold triangle). (**C**) A jackknifed clustering of the environments in the UniFrac dataset (100 permutations). The numbers next to the nodes represent the number of times that particular node was observed (out of 100) in a random sampling from the whole dataset.

Comparison of the individual samples using UniFrac PCA (Figure 4a) showed a distinct clustering by dietary treatment. When the mixed DNA samples were included in the analysis (Figure 4b), they clustered in the center of the individual samples that originated from the same dietary treatment. Jackknife clustering of environments (Figure 4c) showed fairly robust clustering (≥75% bootstrap on all nodes but one) by dietary treatment, similar to the PCA.

**Figure 5:**
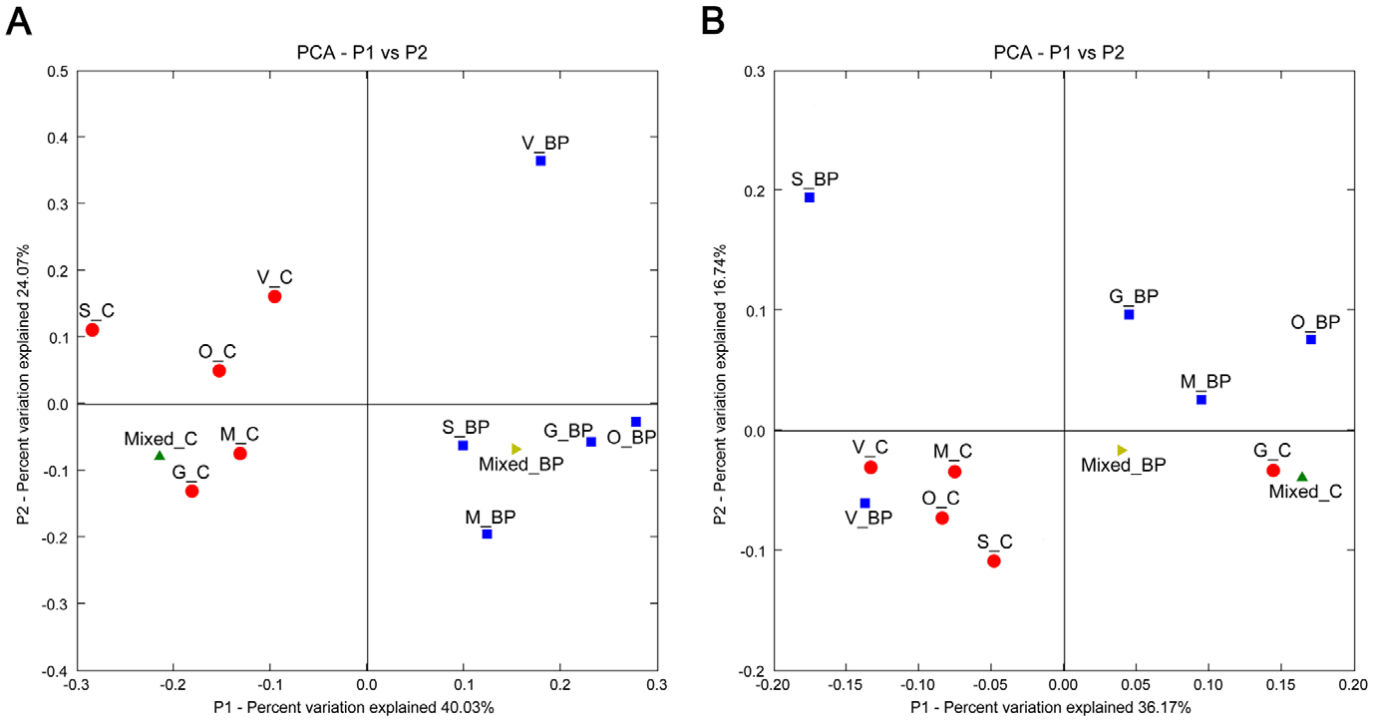
UniFrac analysis of V3 16S sequences (*Firmicutes* and *Fusobacteria* only) from canine fecal samples. Principal Component Analysis scatter plots of individual samples (control  =  red circles; beet pulp  =  blue squares) combined with pooled samples (pooled control  =  green triangle; pooled beet pulp  =  gold triangle). (**A**) Clustering within the phylum *Firmicutes*. (**B**) Clustering within the phylum *Fusobacteria*.

When the phyla that changed significantly based on RDP classifier assignment (Figure 2c) were further evaluated using UniFrac, *Firmicutes* (Figure 5a) appeared to be a strong factor in the clustering of the environments when compared to the *Fusobacteria* (Figure 5b). The clustering based on *Firmicutes* appeared much more defined than that based on *Fusobacteria*. *Actinobacteria* were not further evaluated using UniFrac due to low representation levels and low diversity.

A phylogenetic tree (Figure S1) was constructed for the animal (G) whose gut microbiome structure was among the most affected by diet based on UniFrac analysis. The tree contained 433 total sequences (Sequence Data S3), and showed that the phylogenetic relationship was fairly balanced between the two diets. Notable exceptions were the exclusive appearance of *Eubacterium hallii* on the BP diet, the overrepresentation of *Faecalibacterium prausnitzii* on the BP diet, and the relative overrepresentation of *Fusobacteria* on the C diet.

## Discussion

The dataset presented here is one of the first to utilize “next-generation” sequencing techniques to characterize the canine gut microbiome. Heretofore, most experiments evaluating the dog gut microbiome relied on serial dilution and plating, DGGE, fluorescent in situ hybridization, clone libraries and quantitative PCR, among others. Applying the latest DNA interrogation techniques to companion animal research is necessary to effectively study gastrointestinal health and disease. Whereas the knowledge of the human gut microbiome is rapidly expanding, such knowledge in companion animals is still limited.

In addition to characterizing the canine hindgut microbial communities, we identified changes induced by adding dietary fiber to a high-quality dog food. Beet pulp was chosen as the fiber source because it is commonly used in commercial dog foods and provides a complex mixture of fermentable and non-fermentable carbohydrates. Inclusion of 7.5% beet pulp provides sufficient fermentable substrate to the hindgut, whereas this inclusion level does not depress normal nutrient digestibility [16]. The use of the crossover experimental design allowed us to obtain information on each dog on both diets, essentially letting each dog serve as their own control. No effects of the order in which treatment diets were applied were found, thus generating a powerful experiment with a relatively small number of subjects.

As early as 1977, the number of microbes in the dog hindgut was estimated to be 10^10^ cells per gram of dry contents [17]. If this number is extrapolated to reflect 10 – 100 g dry content present in the hindgut, the total estimated microbial cell number would be between 1 and 10 trillion cells, which approaches estimates in the human gut of 10 – 100 trillion cells [11], [18].

The estimates for microbial diversity in the individual dog gut based on rarefaction of a 2,000-sequence subset noted here is slightly below that in macaques (∼200–∼350 OTU) [19] and approximately similar to diversity reported in humans [20] when using pyrosequencing. In both human and macaque datasets, the sequence number analyzed per sample was approximately similar to those in this experiment (∼1,500–3,000 sequences). Additionally, compared to data in dogs using a near-full-length clone library [12], diversity estimates in the current study are similar. It should be noted that rarefaction analysis based on the full dataset presented here gave substantially higher estimates of OTU (data not shown), particularly in those samples containing larger numbers of sequences.

When evaluating pooled DNA samples based on a 2000-sequence subset, our data suggest an overall number of OTU (96%, Figure 1) that is somewhat lower than that reported in human fecal samples [20] using pyrosequencing. Nevertheless, OTU estimates based on the full datasets for the pooled samples (702 for C and 1,091 for BP) were ∼2–4-fold higher than those reported in humans. Compared to near-full-length 16S clone libraries from human fecal samples [10], [11], diversity in the dog gut appears to be comparable based on data presented here. Although diversity estimates based on OTU observed may differ among host species, a general observation is that even pyrosequencing-based datasets with high read counts do not yet fully cover the complete diversity in gut environments. This is demonstrated here and elsewhere [10], [11], [19]–[21] by rarefaction curves that do not reach saturation. Additionally, non-overlapping confidence intervals for ACE and Chao estimates (Table 1) appear to indicate variable diversity among individuals in the current study. A plausible explanation for these differences is that the current methods do not yet capture the full extent of diversity in the gut. Nevertheless, none of the mammalian gut microbiomes, including that of the dog, appear to be nearly as diverse as the deep sea biosphere [22], [23] or soil communities [24].

The pooled DNA samples appeared to have similar diversity compared to individual samples based on the 2000-sequence subset analysis. Based on full-information analysis, however, diversity estimates for the pooled samples were markedly higher. Nevertheless, the apparent similarity among individual samples and the pooled DNA samples by diet in UniFrac analysis (discussed below) may indicate that the full-information pooled samples accurately reflect the overall community structure as affected by diet. Therefore, pooling DNA samples may give representative results for total community structure in an experiment or experimental treatment, recognizing that pooled data clearly lacks information on individual diversity as was previously noted by Brulc et al. [25]. In many cases individual sampling is likely preferred to obtain maximal information on the individual microbiome response. Nevertheless, in large population studies where the average population response to a treatment is of interest, pooled sampling may decrease cost and analysis complexity.

Whereas diet did not seem to have a major effect on the number of OTU observed in our experiment, the abundance of certain phyla was significantly affected. We detected 7 bacterial phyla, including *Actinobacteria*, *Bacteroidetes*, *Firmicutes*, *Fusobacteria*, *Proteobacteria*, *Spirochaetes*, and *Tenericutes*. However, *Spirochaetes* and *Tenericutes* were not detected in every sample. All of these phyla have been reported in humans [2] and in other species like the chick [26] with the exception of *Tenericutes*. *Tenericutes* have been identified in dogs, but in the small intestine [27]. A striking difference in the dog versus human, macaque, and chick is the apparent dominant presence of *Fusobacteria*. Whereas *Firmicutes* and *Bacteroidetes* typically account for 75% or more of the microbial composition in humans, chicks, and macaques, the dog hindgut appears to be co-dominated by *Firmicutes*, *Bacteroidetes*, and *Fusobacteria*. This observation is supported by previously published data [12], [13] that noted approximately 40% *Firmicutes*, 29% *Fusobacteria*, and 30% *Bacteroidetes* in canine colon contents. Of the less dominant phyla that were present in the current study, the fraction of *Proteobacteria* appeared to be higher than in previously published work (∼7% here vs 1.4%) [12].

Of the three largest phyla *Fusobacteria* appeared to be dominant among them, most notably on the C diet. The addition of fiber to the diet did not greatly alter *Bacteroidetes*, but significantly shifted the *Firmicutes:Fusobacteria* ratio in favor of *Firmicutes*, possibly due to diet selection for complex fermentative activity. This contrasted with earlier findings where dogs fed a fiber-enriched diet (5% soyhulls and 5% beet pulp) did not have different DGGE banding patterns compared to dogs fed a low-fiber diet [14]. When assignments at lower taxon levels (down to genus) are considered, the decrease in *Fusobacteria* was due mainly to lower overall sequence counts, as this phylum was almost exclusively represented by the genus *Fusobacterium*. The dynamics in the *Firmicutes* were more complex, with changes at the class level (Figure 3) but also on lower taxon levels. For example, *Feacalibacterium* (a genus within *Firmicutes*) was tripled (9% to 30%; P<0.05) within the *Firmicutes* when dogs were fed the BP diet. A similar effect was noted on the phylogenetic tree (Supporting Figure S1), where *Faecalibacterium prausnitzii* was more represented on the BP diet. Additionally, the appearance of the butyrate producer *Eubacterium hallii* when dogs were fed the BP diet was not surprising as fermentation activity is likely increased on the BP diet. Taken together, these changes illustrate that the canine gut microbiome can adapt to different dietary components. It should be noted, however, that samples analyzed here provide a snapshot view of gut microbial composition after feeding different diets for at least 10 days. Gradual changes over time in microbiome composition cannot be inferred from the data presented here, as this would require repeated sampling over time. Additionally, changes in composition at lower taxon levels (beyond class) should be carefully interpreted. At lower taxon levels, classification becomes increasingly less reliable with relatively short sequences, and the resolution of our dataset decreased rapidly beyond the higher level taxa. This was the main reason why we were unable to classify most sequences beyond genus level. This loss of resolution at lower levels illustrates that although our dataset is large, it is not yet comprehensive. Choosing different primers spanning a longer or different section of the 16S gene might improve the depth with which the gut microbiome can be evaluated. Moreover, improved chemistry for 454-pyrosequencing now allows for longer sequence reads (∼400 nt) which should improve robustness of classification based on single reads.

The clustering by diet according to UniFrac is striking, taking into account that all samples came from essentially the same environmental conditions, yet a clear separation by diet exists. Previously, in obese mice, UniFrac analysis was able to separate groups by diet fed (a high-fat, high-simple-sugar “western” diet vs. a low-fat, high-polysaccharide diet) [9]. All dogs used here arrived from the vendor together and were housed in a stable environment together for more than one year before conducting this experiment. Within the group of six dogs used, there were three pairs of litter mates; however, no discernable effect of littermates was noted in gut microbial composition unlike reported observations in mice [28].

UniFrac can separate different gut environments efficiently by species such as macaque, human, and mouse [19], but also by diet type (herbivorous, omnivorous, and carnivorous) or gut type (simple, foregut, hindgut) [21]. Furthermore, in silico simulation of pyrosequencing reads generated by using various primers targeting 16S showed that short reads capture the same patterns in diversity as full length 16S sequences, as evaluated by UniFrac [29]. Here, our data suggest that pooled DNA samples represent a robust “average” of the individual samples as illustrated by the placement of the mixed samples in PCA analysis (Figure 4b). This was further supported by the jackknifed environment clustering (Figure 4c), which showed robust (≥75%) bootstrap values for all but one of the nodes.

As expected, UniFrac clustering of *Fusobacteria* gave ambiguous results, which was likely due to the low diversity within this phylum (>99.9% *Fusobacterium*). For the same reason, combined with the low number of sequences classified in the phylum, *Actinobacteria* (who were significantly changed) were not evaluated in this manner. The key phylum that probably was responsible for the distinct clustering by diet was *Firmicutes*, which had a large presence and was highly diverse. This hypothesis was supported by the distinct UniFrac clustering by diet when just sequences from the *Firmicutes* were analyzed (Figure 5a).

In summary, we show here that the composition of the dog gut microbiome was successfully interrogated with 454-pyrosequencing using barcoded primers to amplify segments of the 16S rDNA gene. Our results on bacterial diversity are in agreement with data published from gut microbiomes of other species, and the classified composition of the bacterial community is congruent with data reported in dogs using clone library techniques. It should be noted, however, that the approach used here can and should be further refined to obtain a higher resolution. This will allow for studying the dog gut microbiome at a deeper level than was possible here. Last, we show that a relative small amount of dietary fiber gives rise to a significant and detectable change in the composition of the gut microbial communities, contrasting a previous investigation in dogs using DGGE. These changes may be of importance because of emerging evidence that distinct differences exist between “healthy” and “diseased” gut microbial communities [5], [10], [30]. Identifying specific dietary effects on the gut microbiome will allow for targeted and effective dietary interventions for the alleviation of microbiome-associated maladies.

## Materials and Methods

### Animals and diets

All animal care procedures were approved by the University of Illinois Institutional Animal Care and Use Committee prior to initiation of the experiment. Six female healthy adult (mean age  = 20 mo; mean bodyweight  = 20.3 kg) purpose-bred dogs (two mongrels, four hound-crosses; Marshall Bioresources, North Rose, NY) were used in a crossover experimental design. None of the dogs used were obese. Animals were housed under environmentally controlled conditions (22°C, 12 h light-12 h dark cycle) at the Small Animal Clinic of the University of Illinois College of Veterinary Medicine.

The experimental diets (Table 2) were formulated to contain approximately 30% crude protein and 20% fat. The main ingredients were brewer's rice and poultry byproduct meal. The control diet (C) contained no supplemental dietary fiber, whereas the fiber-supplemented diet (BP) had 7.5% beet pulp (60% total dietary fiber, ∼4∶1 insoluble:soluble fiber) added, replacing 7.5% brewer's rice. The diet formulation was milled at Lortscher Agri Service, Inc. (Bern, KS) and extruded at Kansas State University's BIVAP facility (Manhattan, KS) under the direction of Pet Food and Ingredient Technology, Inc. (Topeka, KS).

**Table 2:** Ingredient and nutrient composition of diets fed to adult dogs to evaluate gut microbiome composition.

Item, %	Control diet	Beet pulp diet
	-----------------As-is basis-----------------	
Brewer's rice	45.22	37.72
Poultry byproduct meal	37.00	37.00
Poultry fat	14.00	14.00
Beet pulp^1^	0.00	7.50
Dried egg	2.40	2.40
Potassium chloride	0.56	0.56
Salt	0.45	0.45
Choline chloride – 50%	0.13	0.13
Vitamin premix^2^	0.12	0.12
Mineral premix^3^	0.12	0.12
Dry matter	94.62	95.06
	-------------Dry matter basis--------------	
Organic matter	93.21	92.85
Crude protein	29.72	28.04
Acid hydrolyzed fat	19.41	20.97
Total dietary fiber	1.39	4.49
Soluble dietary fiber	0.91	1.82
Insoluble dietary fiber	0.48	2.67

1 Beet pulp: 60.27% total dietary fiber; 12.24% soluble dietary fiber; 48.03% insoluble dietary fiber.

2 Provided per kilogram of diet: Vitamin A, 10560 IU; vitamin D_3_, 1056 IU; vitamin E, 105.6 IU; vitamin K, 0.5 mg; thiamin, 2.6 mg; riboflavin, 3.4 mg; pantothenic acid, 13.2 mg; niacin, 23.8 mg; pyridoxine, 2.1 mg; biotin, 0.1 mg; folic acid, 264 µg; vitamin B_12_, 66 µg.

3 Provided per kilogram of diet: Manganese (MnO), 66 mg; iron (FeSO_4_), 120 mg; copper (CuSO_4_), 18 mg; cobalt (CoCO_3_), 1.2 mg; zinc, (ZnO), 240 mg; iodine (C_2_H_6_N_2_·2HI), 1.80 mg; selenium (Na_2_SeO_3_), 240 µg.

### Animal experimental procedures

The experiment used a crossover design with two 14-d periods. Dogs were randomly assigned to one of the two experimental diets prior to the first period, and subsequently received the second diet in period two, so that each animal served as its own control. Dogs were fed once daily and 300 g of the assigned diet was offered. This amount was sufficient to meet the metabolizable energy needs of the heaviest dog based on NRC recommendations [31]. At each feeding, leftovers from the previous feeding were collected and weighed. After a 10-d diet adaption phase, a fresh fecal sample was collected during the next 4 d for DNA extraction.

### Sample handling

Immediately after feces were voided, they were collected, weighed, aliquoted into cryogenic vials (Nalgene, Rochester, NY) and then flash-frozen in liquid nitrogen. After the samples were thoroughly frozen, they were stored at −80°C until DNA extraction.

### DNA extraction and PCR procedure

DNA was extracted using a modification of the method of Yu and Morrison [32]. Briefly, sterile glass beads (0.1 g of 0.5 mm and 0.3 g of 0.1 mm; Biospec Products, Inc., Bartlesville, OK) were added to each ∼200 mg fecal sample to facilitate the breaking of the fecal sample by vortexing. The aggressive bead-beater steps were skipped to reduce DNA shearing. After extraction, DNA was quantified using an ND-1000 spectrophotometer (Nanodrop Technologies, Wilmington, DE) to ensure that total DNA yield was at least 5 µg. An aliquot of the DNA then was diluted to 50 ng/µL prior to use in PCR.

Amplification of the variable region 3 of the bacterial 16S rDNA gene was utilized to assess gut microbial diversity. Primers used to amplify the V3 region (341F and 534R) [33] have been widely used in DGGE. To allow for the use of the PCR product in 454 pyrosequencing, fusion primers (IDT, Coralville, IA) were designed that contained the adapters required for this procedure. In addition, these primers also contained a 10 nt barcode sequence that allowed for multiple samples to be analyzed in a single sequencing run.

Initial PCR was performed in triplicate for each sample in a total reaction volume of 50 µL. Each 50 µL reaction mixture contained 1.25 U Takara Ex Taq DNA polymerase, 5 µL 10X Ex Taq buffer (Mg^2+^), 4 µL dNTP mix (all Takara Bio USA, Madison, WI), 10 pmol each of the forward and reverse primer, 1 µL (∼50 ng) of extracted DNA, and was brought to 50 µL using sterile water. To minimize PCR bias (where highly represented sequences are amplified faster than those that are rare), we used 20 PCR cycles and obtained sufficient quantities of product. The PCR conditions were as follows: initial denaturing, 94°C for 5 min followed by 20 cycles of 30 s at 94°C (denaturing), 30 s at 69°C (annealing), 30 s at 72°C (extension), and after cycling was complete, 7 min at 72°C to extend unfinished product. After PCR, the resulting product was checked for size and purity on an agarose-EtBr gel and then prepared for 454-pyrosequencing according to manufacturer instructions with the deviation that the PCR product was cleaned using a Qiagen silica column (Qiagen, Valencia, CA) instead of sizing beads. In addition to individual DNA samples, two pooled (by diet) DNA samples were prepared, such that each individual sample contributed an equal amount of DNA. These pooled samples were then amplified and sequenced to assess the effect of sample pooling on sequencing results. Sequencing of the PCR products was performed at the W. M. Keck Center for Biotechnology at the University of Illinois using a 454 Genome Sequencer FLX (Roche Applied Science, Indianapolis, IN). After sequencing was completed, all reads were scored for quality and any poor quality reads and primer dimers were removed. All sequences that passed quality control are provided by sample in Sequence Data S1 (sequences derived from C samples) and Sequence Data S2 (sequences derived from BP samples).

### Data analysis

To assess bacterial diversity in the DNA samples in a comparable manner, a randomly selected, 2,000-sequence subset from each sample was aligned with MUSCLE [34] using the *–maxiters 2* option. A distance matrix was calculated from the alignment with PHYLIP [35] using the kimura-2 correction option. Operational Taxonomical Units (OTU; 96% identity) then were assigned by DOTUR [36] with default options. To build a tree to outline phylogenetic relationships between diets in a single animal based on the total number of sequences obtained, representative sequences for each OTU were selected using the get.oturep command in Mothur 1.5 [37]. A tree was built using the neighbor-joining method of Clustal_X [38] with 1000 bootstrap replicates. The tree was displayed and edited using the Molecular Evolutionary Genetics Analysis package (MEGA4) [39]. Representative sequences were searched using NCBI BLAST, and the nearest matched species (≥90%) was assigned.

UniFrac was used to evaluate the relatedness of samples within the same dietary treatment[40], [41]. Because of the large sequence number in the dataset, we grouped similar sequences (96% identity) together picking one representative for a group using Fastgroup II [42]. Sequences shorter than 59 nt were excluded from subsequent analysis. Representative sequences from all samples were aligned together using MUSCLE, a distance matrix was calculated using PHYLIP, and a single tree was built using Clearcut [43]. This tree served as the input tree for UniFrac. Weighted and normalized Principal Component Analysis (PCA) was performed to evaluate similarity among samples, where each sample represents an environment.

Evaluation of represented bacterial phyla in dog feces was done using the classifier of the Ribosomal Database Project (v10.2) [44]. The deepest taxa assignment for each sequence with >80% confidence was used to assess the composition of the bacterial population. Relative changes in microbial sequences between the diets were analyzed statistically using the Mixed procedure of SAS (SAS Institute, Cary, NC), and P<0.05 was considered significant. Sequences that represented phyla that changed with dietary treatment then were analyzed using UniFrac as described above to evaluate their similarity or dissimilarity as affected by diet.

## Supporting Information

Figure S1 Phylogenetic tree constructed from sequences from one dog (G). The tree displays phylogenetic relationships between the microbiome structures when the dog was fed two different diets. Sequences marked red are from the control diet, sequences in black are from the beet pulp-supplemented diet.
(0.08 MB PDF)

Sequence Data S1 Sequences derived from dogs fed the Control diet, individual animals (5) and a pooled DNA sample.
(0.73 MB ZIP)

Sequence Data S2 Sequences derived from dogs fed the Beet pulp diet, individual animals (5) and a pooled DNA sample.
(0.61 MB ZIP)

Sequence Data S3 Representative sequences selected from dog G data used to construct the tree in Figure S1. First 2 digits of the sequence tag indicate the source sample of the sequence (CO: Control; BP: Beet pulp). After the 8-digit unique sequence string, |x|y indicate the OTU number in the original sample (x) and the number of sequences in that particular OTU (y).
(0.01 MB ZIP)
