# Supplementary figures and images for: Phylogenetic Characterization of Fecal Microbial Communities of Dogs Fed Diets with or without Supplemental Dietary Fiber Using 454 Pyrosequencing
Source: PLoS One. 2010 Mar 22;5(3):e9768. doi: 10.1371/journal.pone.0009768 (PMC2842427; doi:10.1371/journal.pone.0009768)

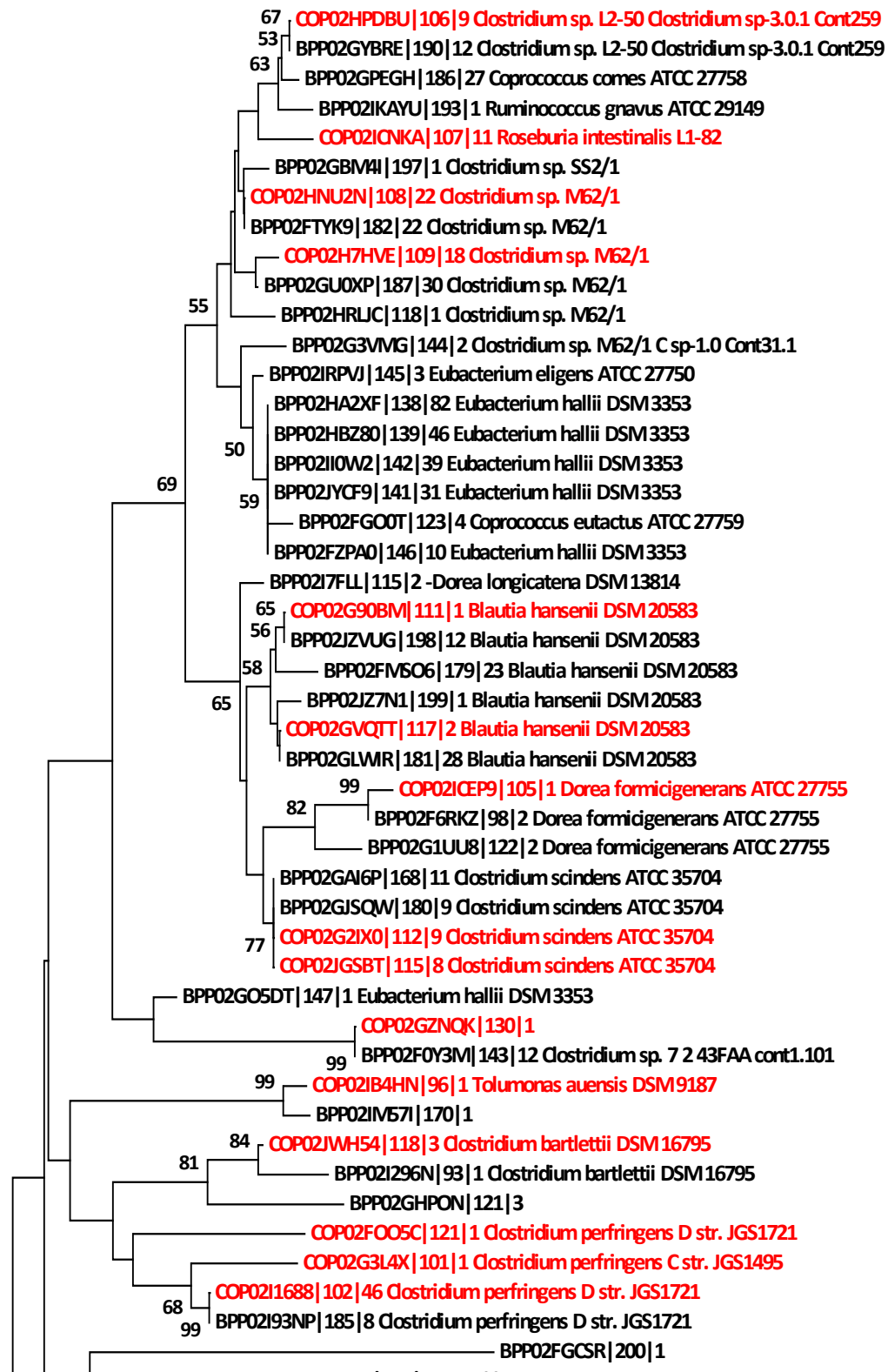

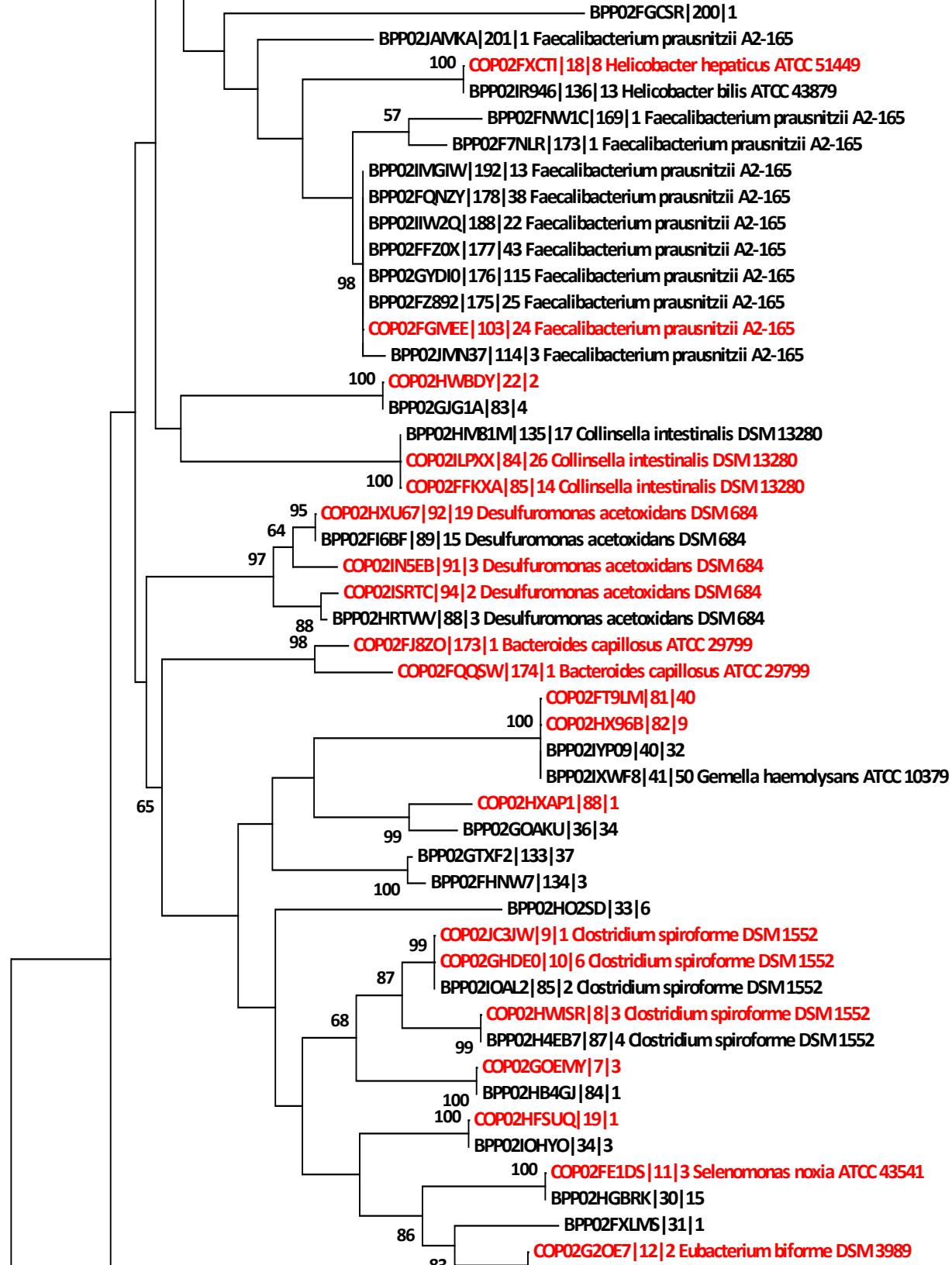

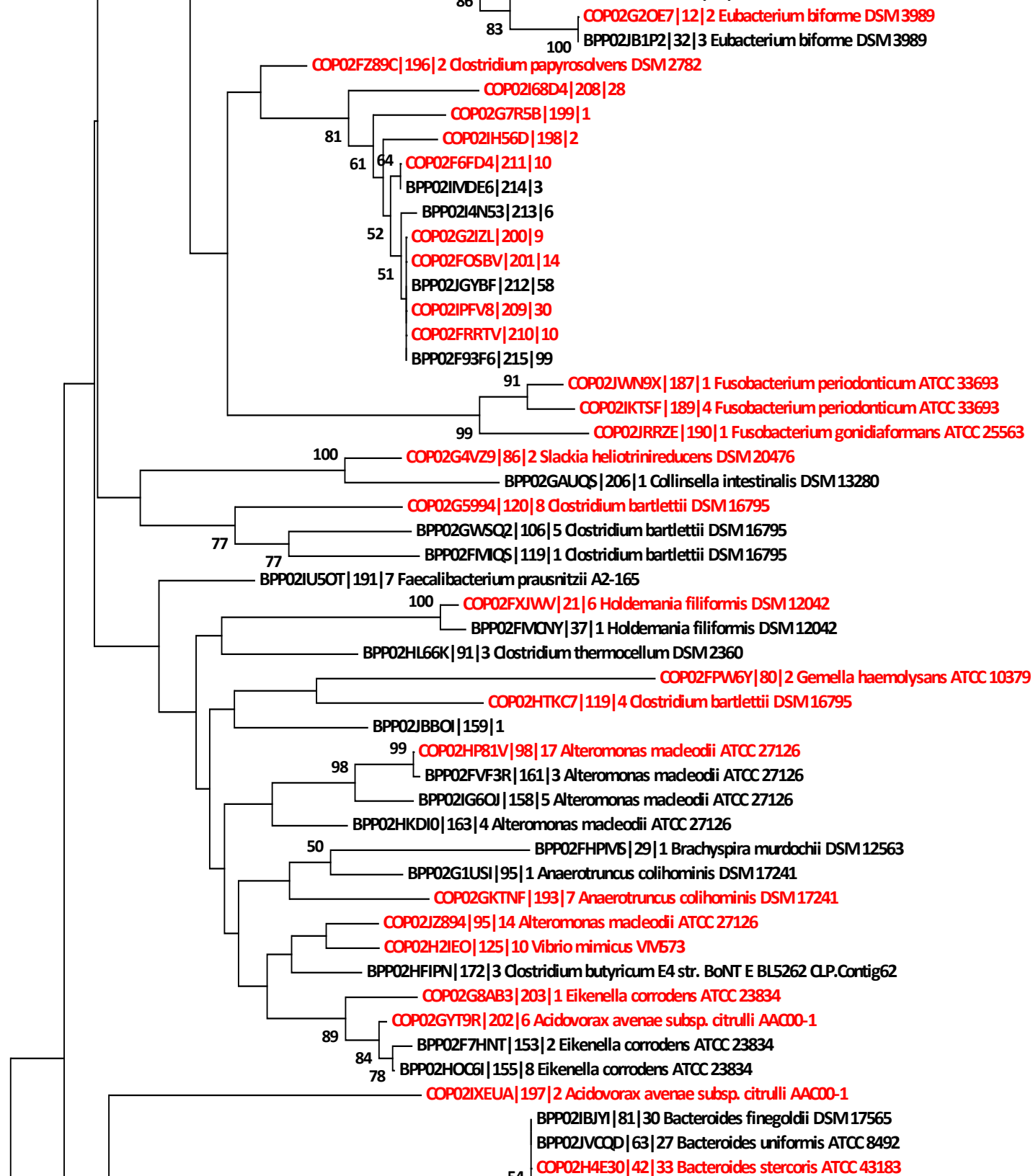

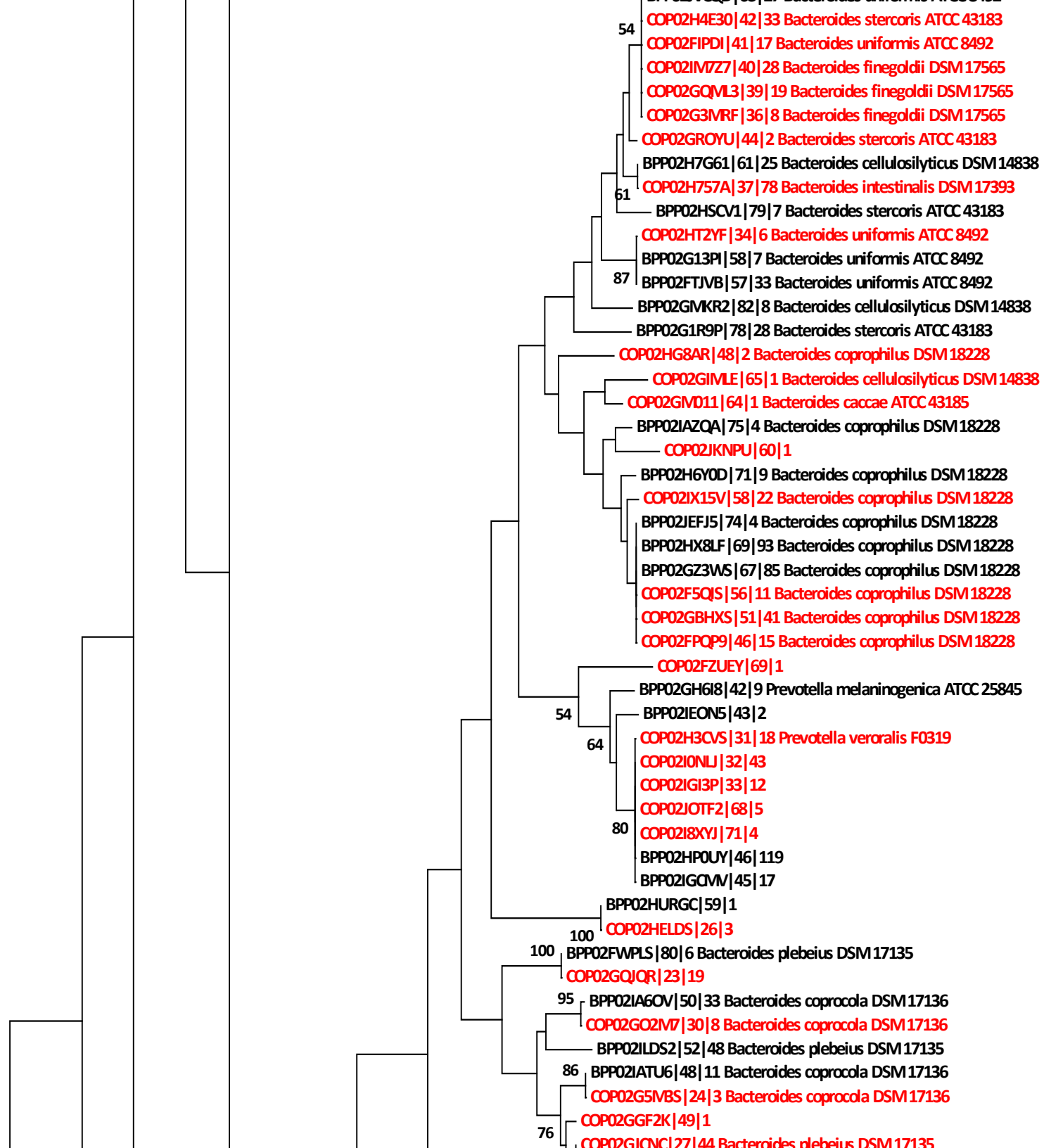

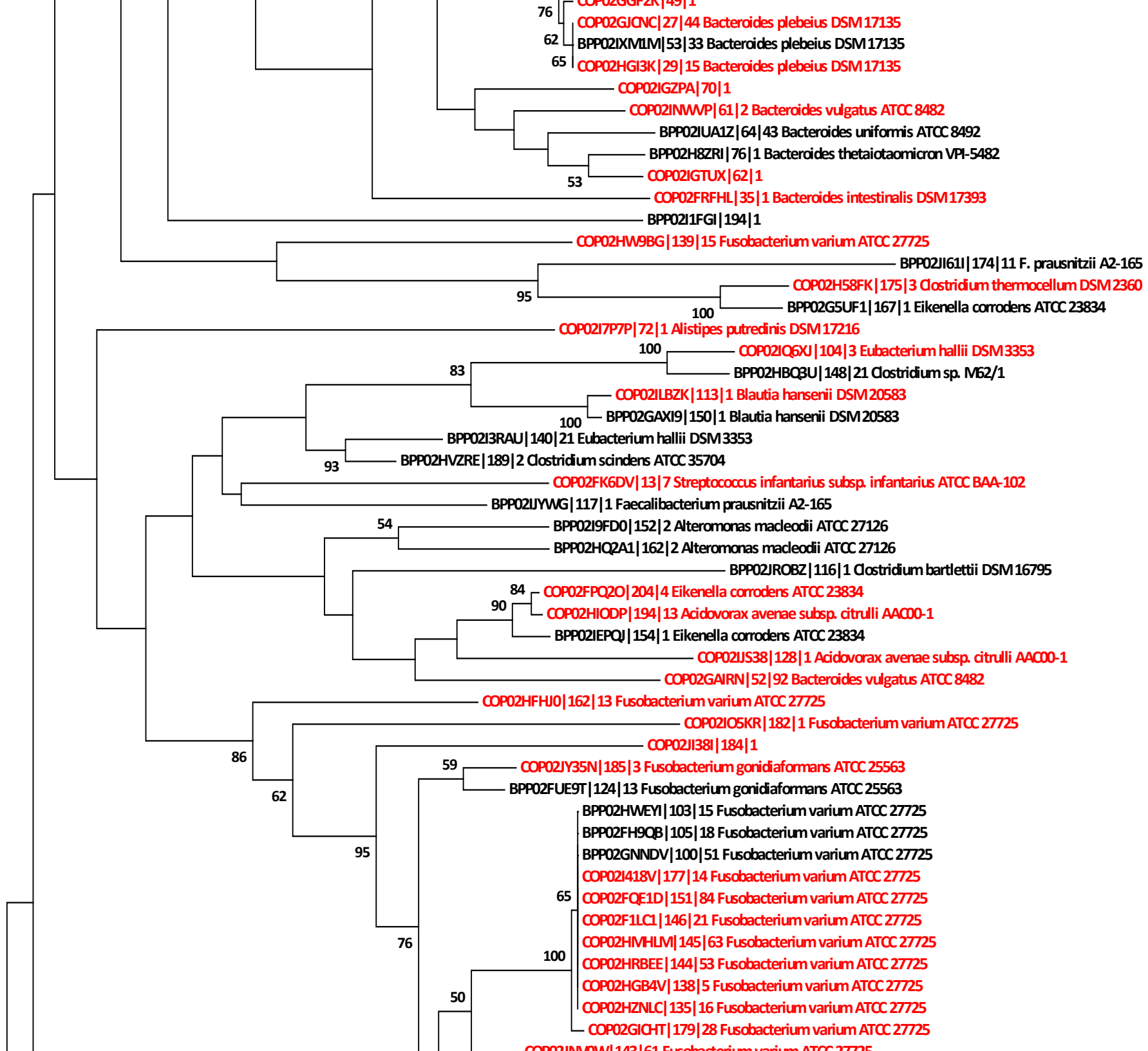

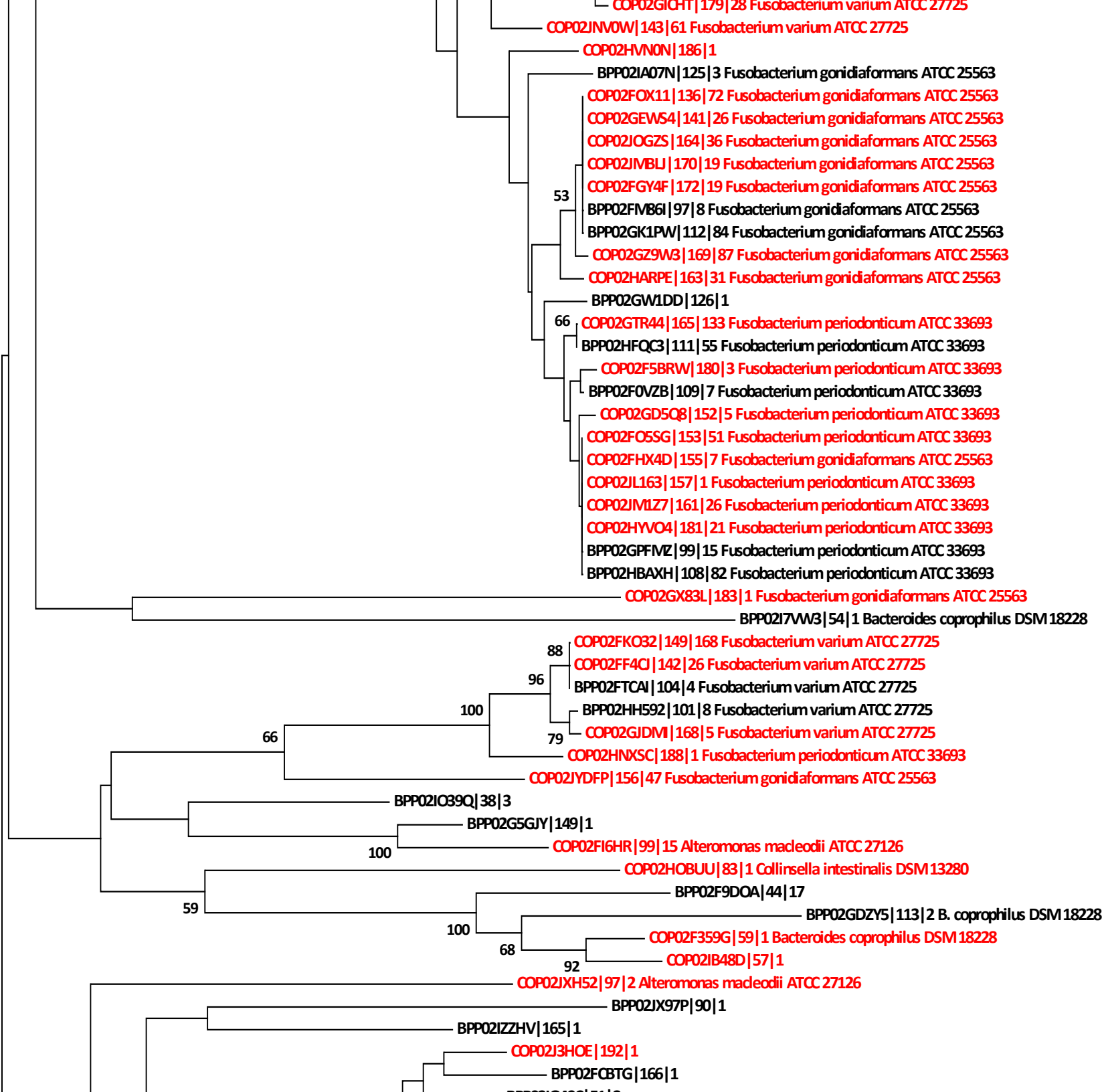

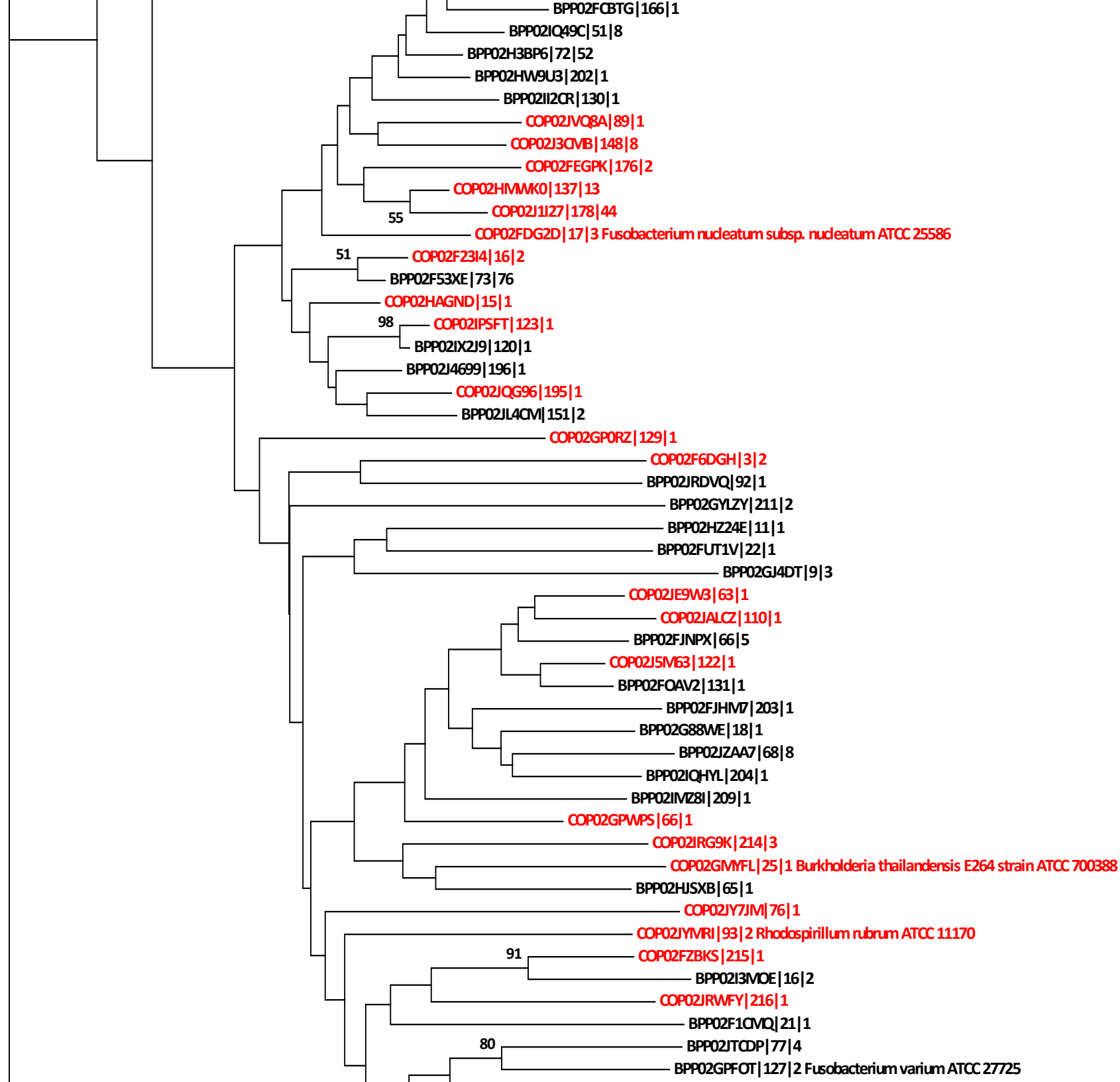

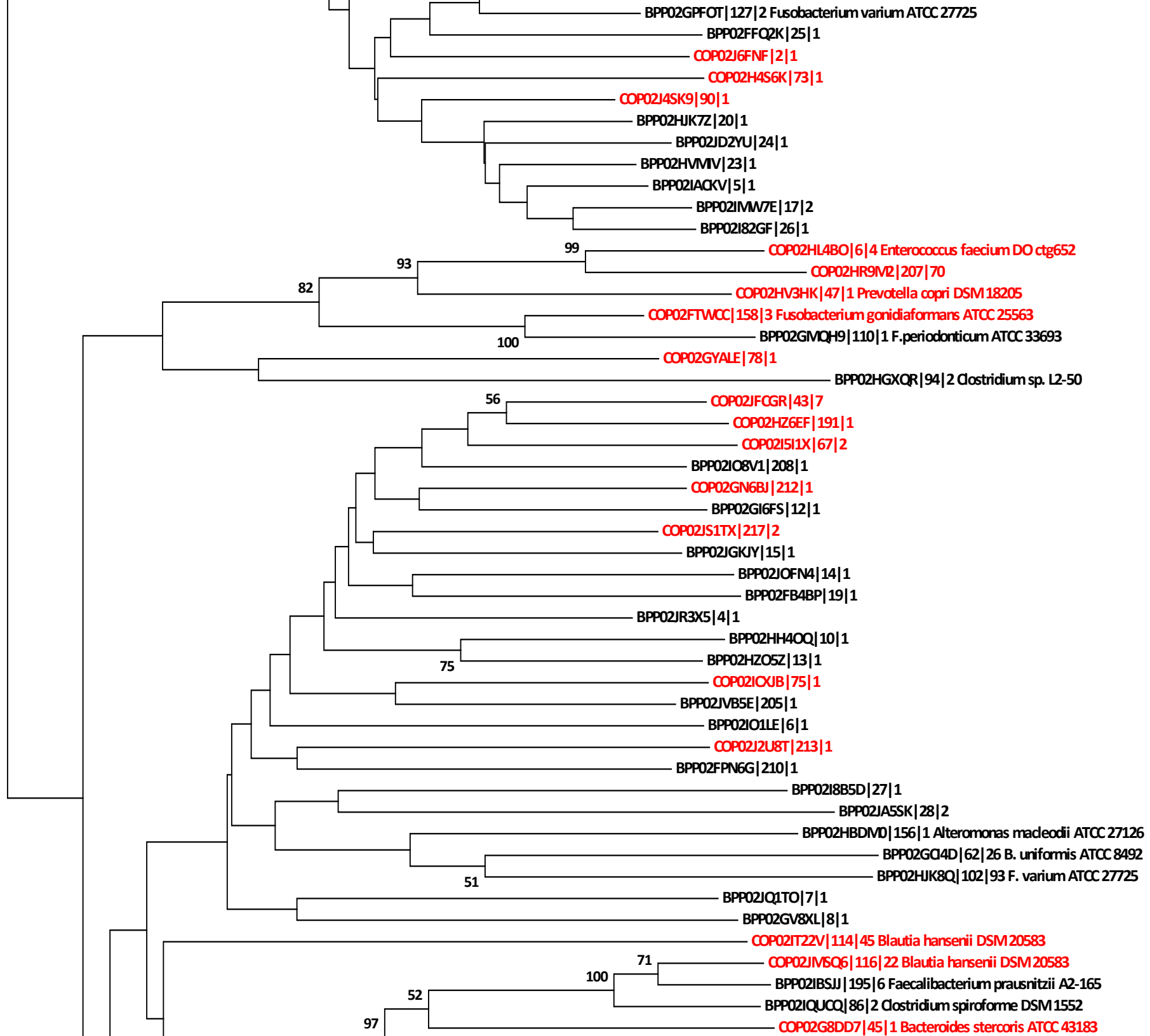

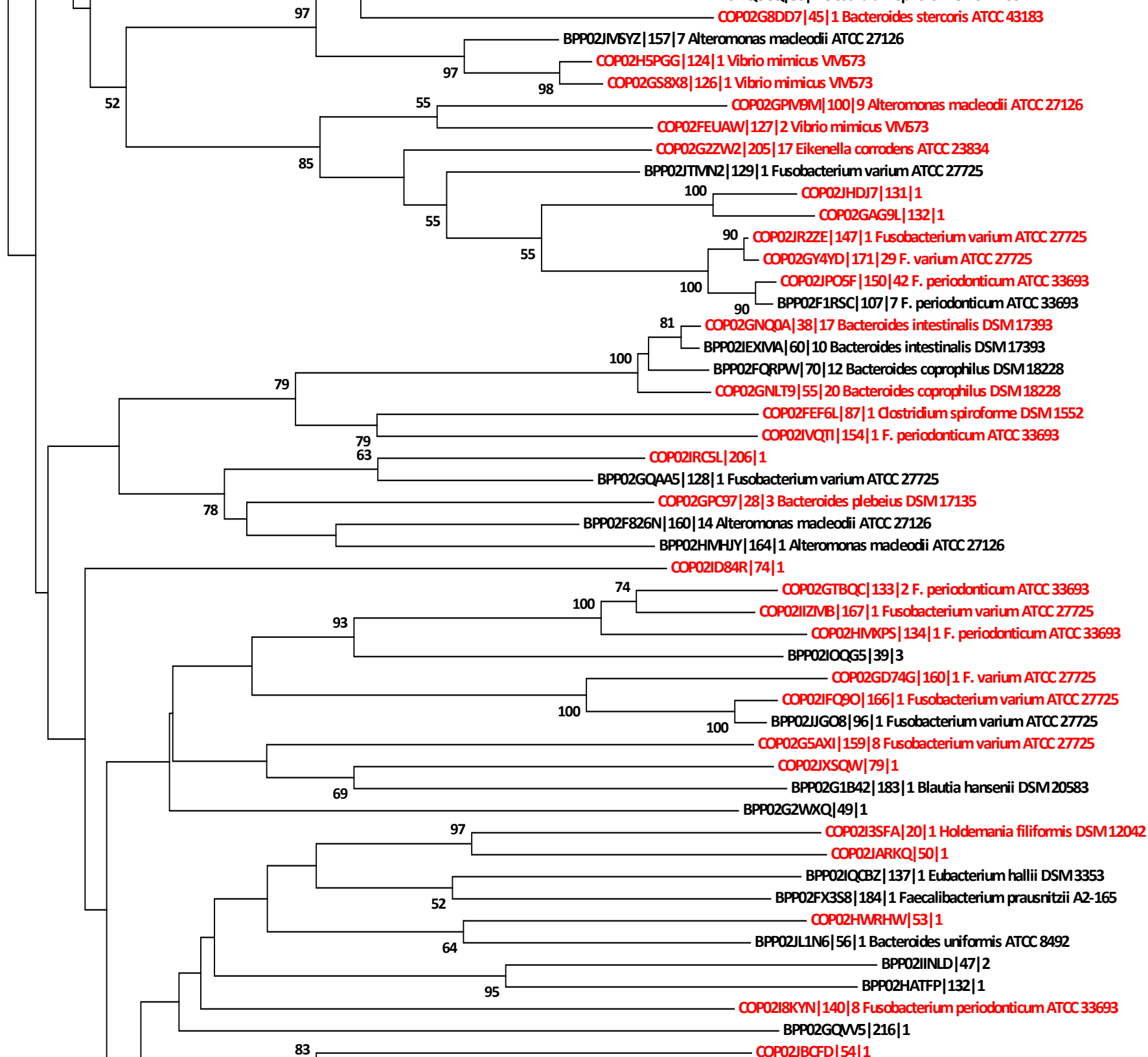

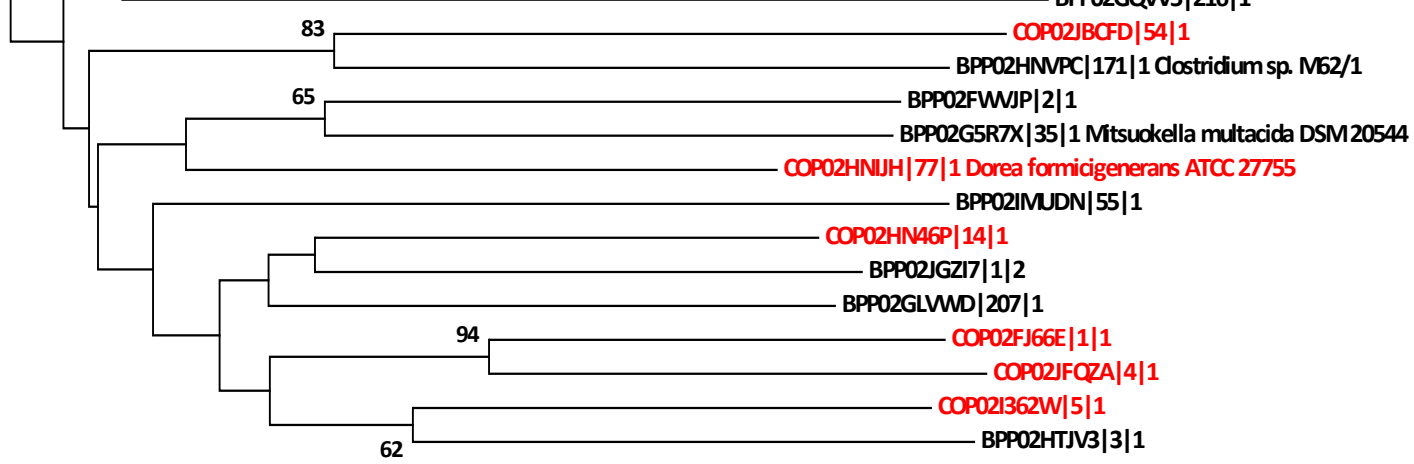

0.05

Supplement: Figure S1 — Phylogenetic tree constructed from sequences from one dog (G). The tree displays phylogenetic relationships between the microbiome structures when the dog was fed two different diets. Sequences marked red are from the control diet, sequences in black are from the beet pulp-supplemented diet. (0.08 MB PDF) [file pone.0009768.s001.pdf]
